# Increases in the Association Between the Rates of Synchronous and Metachronous Metastases over Time †

**DOI:** 10.3390/jcm14082762

**Published:** 2025-04-17

**Authors:** Ugur Yilmaz, Steven P. Rowe, Lawrence B. Marks

**Affiliations:** 1 Department of Radiation Oncology, Kartal Dr. Lutfi Kirdar City Hospital, Istanbul 34865, Türkiye; druguryilmaz@gmail.com; 2 Department of Radiology, Lineberger Cancer Center, University of North Carolina at Chapel Hill, Chapel Hill, NC 27599, USA; steven_rowe@med.unc.edu; 3 Departments of Radiation Oncology, Lineberger Cancer Center, University of North Carolina at Chapel Hill, Chapel Hill, NC 27599, USA

**Keywords:** synchronous, metachronous, metastasis

## Abstract

**Background:** This study investigates the association between synchronous and metachronous metastases across various cancer types, evaluating whether that relationship has evolved over time. **Methods:** Data from the Surveillance, Epidemiology, and End Results (SEER)-8 dataset from 1975 to 2020 were retrospectively reviewed. For each of the 18 solid tumor types, the crude rates of synchronous and metachronous metastases were estimated from the SEER database. For each of the years assessed (from 1975 to 2015 at 10-year increments), linear regression analyses were conducted to quantify the relationship between the rates of metachronous metastasis and synchronous metastasis across all cancer sites. The *degrees* of association over time were compared using a Fisher’s z-transformation. **Results:** At all time points considered, there was a significant association between the rates of metachronous and synchronous metastases (all *p* values < 0.05 for 5-year follow-up data). The *degree* of that association tended to increase over time (R = 0.59, 0.60, 0.66, 0.80, and 0.87 for 1975, 1985, 1995, 2005, and 2015, respectively), with the *p* value of the z-score comparing the many R values over time varying from 0.04 to 0.48. **Conclusions:** There appears to be an increasing association between the rates of synchronous and metachronous distant metastases over time. The exact cause of this increasing association is unknown. However, it appears to have occurred somewhat contemporaneous with the increasing use of more-accurate imaging studies (e.g., FDG-PET). Newer, targeted radiotracers for PET may provide the landscape for a prospective evaluation of the role of imaging.

## 1. Introduction

At diagnosis, the staging of cancer describes the extent of identifiable disease, and generally guides therapy and provides prognostic information. Imaging plays an important role in staging. Nevertheless, it is generally understood that imaging and staging are imperfect; e.g., some patients without *evidence* of distant metastasis at diagnosis actually do harbor subclinical distant metastases [1,2]. Indeed, the development of overt metastases relatively soon after initial diagnosis is a manifestation of this reality, and the widespread use of adjuvant systemic therapy in many settings is an acknowledgement of that reality.

It is customary to categorize (albeit imperfectly) distant metastases to be either synchronous (present at, or very soon after, the time of diagnosis) or metachronous (being detected sometime later). Since different tumors have different biological behaviors, and are often staged using very different approaches (e.g., physical exam, surgical staging, and imaging being of variable utility in different body sites), it is not surprising that different tumors have markedly variable *absolute rates* of synchronous and metachronous distant metastases.

Nevertheless, our prior analyses suggest that the estimated *fraction* of all distant metastases that are synchronous vs. metachronous appears to be *somewhat* similar across different cancer types [3,4]. This might not be too surprising since somewhat similar approaches have been taken for decades, across different tumor types, to evaluate patients for the presence of distant metastases based on clinical symptoms (e.g., bone pain, weight loss, shortness of breath, headache), physical examination (e.g., palpation of node-bearing areas and liver, bone tenderness, etc.). Furthermore, similar imaging tools have played a central role in the diagnosis of metastases across different types of cancer (e.g., chest X-ray, myelograms, plain films, and lymphangiograms many decades ago; evolving to bone scan, axial CT somewhat later, and more recently to magnetic resonance [MRI] and positron emission tomography [PET]). However, the relative utility of these various clinical, physical exam, and imaging-based assessments certainly varies across tumor sites, and thus it is not surprising that the previously noted associations between the rates of synchronous and metachronous metastases across different types of cancer are relatively weak [3]. On the other hand, gradual improvements in staging (e.g., via better imaging, laboratory, or other means) may, over time, improve this association.

Understanding the association between the observed rates of synchronous vs. metachronous metastases, across tumor types, has practical implications. Broadly, it can provide an improved *quantitative* understanding of the limitations of current staging methods. That could (1) improve providers’ abilities to better contextualize the results of staging studies to patients, and (2) hopefully enable patients to better appreciate the limitations of their own test results, the uncertainties in the estimates of their own prognosis, and help them to better understand why some treatments are recommended. For example, patients often seem confused by the apparent contradiction between, “your test results all look good”, and “we are recommending additional treatment”.

In addition, if there were a well-understood relationship between the rates of synchronous vs. metachronous metastases, one could estimate the anticipated expected rate of metachronous metastases (which is often unknown) based on the observed rate of synchronous metastases (which is far easier to assess). This might help us to better understand whether adjuvant therapies are altering the natural history for a new/evolving form of cancer. For example, in some settings (especially for uncommon diagnoses), patients may accept adjuvant therapies, and it is challenging to know whether this was helpful. Comparing the observed rate of metachronous metastases to the estimated expected rate of metachronous metastases (based on the rate of observed synchronous metastases), one may be able to assess whether these adjuvant therapies were helpful. The “gold standard” approach to assess the utility of adjuvant therapy (i.e., the prospective randomized clinical trial) is often not possible nor practical (e.g., due to logistical issues, expense, etc.).

Our initial analysis of SEER (Surveillance, Epidemiology, and End Results) data from 1973 to 1998 noted a weak association between the rates of synchronous and metachronous metastases across different cancer types, and no meaningful changes in this associations over the years considered [3]. Our subsequent analysis of SEER data from 1973 to 2008 noted subtle increases in the fraction of distant metastases that were synchronous, in some tumor sites, over time [4]. In this current analysis, we again assess if the association between the rates of synchronous and metachronous metastases across different types of cancer has changed over time, but now with analyzable SEER data available through 2015 (i.e., over a much longer time frame than in our prior analysis [3]). We hypothesize that this association has become stronger over time, perhaps due to improvements in staging.

To test our hypothesis, we used updated population data from SEER. However, SEER and similar databases do not specifically report the incidence of metachronous metastases. Thus, we employed a transparent approach to estimate the rates of both synchronous and metachronous metastases from SEER, and determined how the association between these estimates of synchronous and metachronous metastases changed over time (rather than attempting precise measures of those rates of metastases).

## 2. Material and Methods

### 2.1. Patient Population and Data Extraction

This retrospective study was conducted in accordance with the guidelines of the Declaration of Helsinki.

Analyses were performed by using the SEER-8 dataset from 1975 to 2020, consisting of eight cancer registries across the United States. SEER*Stat Version 8.4.2 software was used for data extraction [5,6]. The “frequency” application was used to calculate crude rates, and the “survival” application was used to calculate cancer-specific death rates [7], as described below.

SEER historic stage A, which was recorded consistently from 1975 to 2015, was used for the analysis. The cancer sites were chosen according to the Site Recode ICD-0-3/WHO 2008.

All anatomic sites reported by SEER were considered for inclusion. The following sites were excluded: Hematological malignancies were excluded due to their presentation essentially always as systemic; Ovarian cancer was excluded due to its pattern of intra-abdominal dissemination (and thus discordance in how stage is defined compared to most other tumor sites); and several others were excluded due to consistently small numbers of evaluable cases over the years considered (i.e., <250 cases/year), and often with highly variable cancer death rates in consecutive years due to these small numbers.

For each of the remaining 18 solid tumor types (colon, rectum and rectosigmoid junction, esophagus, oral cavity and pharynx, corpus and uterus, cervix uteri, breast, pancreas, kidney and renal pelvis, stomach, melanoma of the skin, urinary bladder, testis, thyroid, soft tissue including heart, larynx, lung and bronchus, and prostate), the crude rates for several metrics were extracted for patients diagnosed in each year from 1975 to 2015. The SEER data are complete across all 18 tumor sites, with the exception of incomplete data for lung and bronchus cancer from 1975 to 1987, for prostate cancer from 1975 to 1994, and for larynx cancer after 2003, and those specific data points were thus not available to be included in the analyses.

### 2.2. Estimating the Rates of Synchronous and Metachronous Distant Metastatic Disease

#### 2.2.1. Synchronous

The proportion of patients with distant metastatic disease at the time of initial diagnosis is taken as the ratio of distant cases to the sum of localized, regional, and distant cases reported in SEER (distant/[localized + regional + distant]).

We acknowledge that there might be a fraction of patients who are inaccurately categorized as having metastases at diagnosis (e.g., a false positive finding on imaging), but submit that this fraction is likely small; e.g., partly on the basis that many imaging findings that would suggest solitary or low-volume metastatic disease would generally be histologically investigated before any final treatment decisions would be made.

#### 2.2.2. Metachronous

Since the majority of cancer deaths are due to distant metastases, we use death as a surrogate for metastases. The fraction of patients developing metachronous metastases (at 5, 10, and 15-years post-diagnosis) is estimated as the product of (The fraction of patients presenting with localized and/or regional disease [clinical M0]) * (The 5-year, 10-year, and 15-year cancer-specific death rates in patients presenting with localized and/or regional disease).

**Figure 1:**
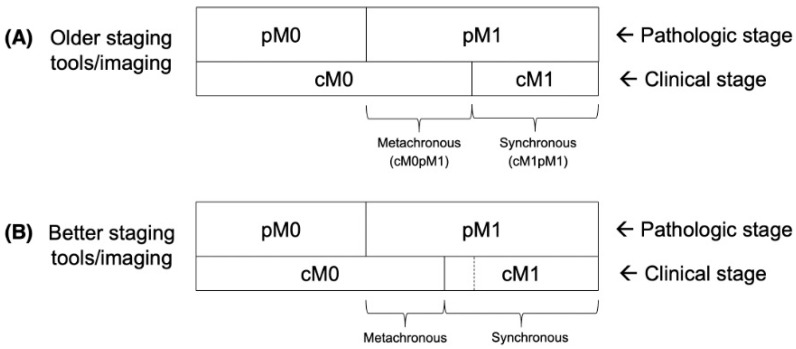
Idealized diagram (not to scale) illustrating pathologic stage (pM0 vs. pM1) and clinical stage (cM0 vs. cM1) migrations among older staging tools/imaging vs. better staging tools/imaging. The border between cM0 and cM1 in the panel (**A**), with older staging tools/imaging, is represented by a dashed line in panel (**B**). Additional scenarios including better adjuvant/therapeutic systemic therapy, better screening (earlier detection), and some combinations, are provided in Supplementary Figure S1. Images are adapted from our prior analyses, Anacak [3] and Yilmaz [4].

Figure 1 demonstrates the association between different clinical and pathological stages at diagnosis. To assess for changes over time, this calculation was repeated for patient cohorts from 1975, 1985, 1995, 2005, and 2015. Follow-up intervals of 5-, 10- and 15-years were considered (with the more recent cohorts not having longer-term follow-up data available).

In the formulation we defined, it is assumed that the majority of patients developing metachronous metastases harbored subclinical metastases at diagnosis. We acknowledge that some patients can develop distant disease after initial diagnosis (e.g., from uncontrolled localized/regional disease) but submit that this is relatively uncommon [8]. We also acknowledge that some patients can die from uncontrolled localized/regional disease, and not from distant metastases, but submit that this is also relatively uncommon, and is only a major issue for some specific diagnoses (e.g., cancers of the head and neck and cervix). That possibility is considered by repeating the analyses with these cancer types excluded. Furthermore, since we are assessing for *changes* in the association between the rates of synchronous and metachronous metastases *over time*, we believe that our approach is reasonable since we are using this same (albeit imperfect) measure over time.

### 2.3. Statistical Analysis

For each patient cohort (1975–2015), and for each follow-up interval (5- 10-, and 15-years), simple linear regression analyses were performed to quantify the relationship between the rates of metachronous metastases (dependent variable) and synchronous metastases (independent variable) across all cancer sites considered. The distribution of normality for dependent variables was determined by Shapiro–Wilk test (*p* > 0.05).

Correlation coefficients (R) and regression coefficients (slope) were computed. Fisher’s z-transformation was applied to assess the significance of the difference between any two of the R values. *p* < 0.05 was considered statistically significant. Statistical analyses were conducted using R version 4.3.2 (R Foundation for Statistical Computing, Vienna, Austria).

## 3. Results

The data extracted from the SEER registry are provided in Supplementary Table S1. Regression statistics for 5-year, 10-year, and 15-year data across all cancer sites considered are included in Table 1.

**Figure 2:**
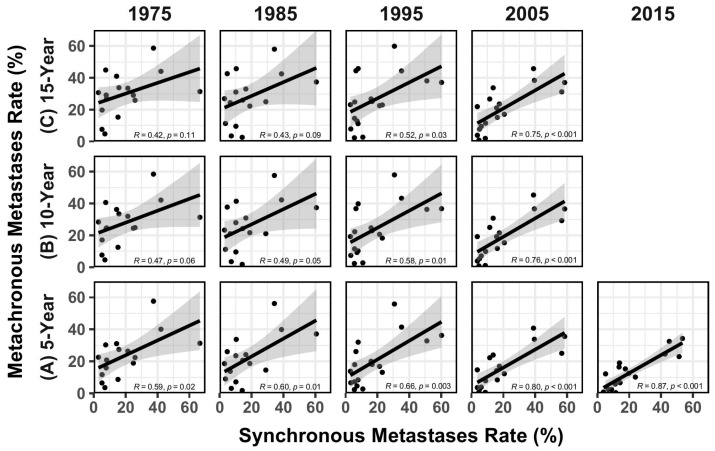
Regression analysis between the rates of synchronous metastases rate and metachronous metastases rate based on (**A**) 5-year, (**B**) 10-year, and (**C**) 15-year survival data from 1975 to 2015 (the data are missing for lung and bronchus cancer, and prostate cancer in 1975 and 1985, and the data are missing for larynx cancer in 2005 and 2015). Gray areas around the lines represent the standard error of the regression line.

**Figure 3:**
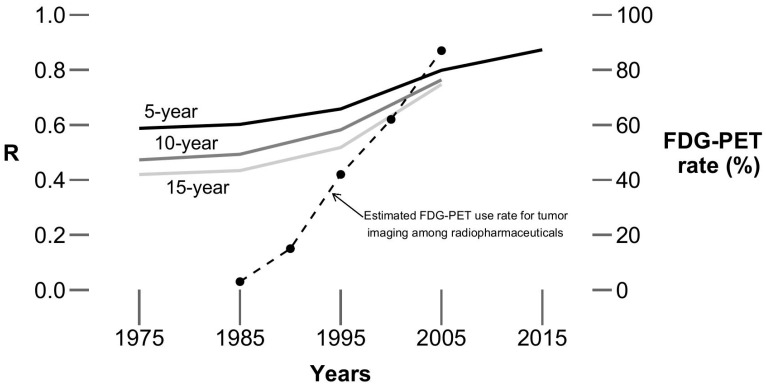
R values over time. Solid lines depict the change in R values (the data include all (18) cancer sites), estimated by 5-, 10-, and 15-year survival data, over time (from 1975 to 2015, by 10-year intervals). The dashed line indicates estimated FDG-PET use rate for tumor imaging among radiopharmaceuticals (from Drozdovitch et al.) [9]. The timing of the marked increases in the R values corresponds roughly to the timing of increases in the use of FDG-PET.

The association between rate of synchronous and metachronous metastases (based on 5-year, 10-year, and 15-year survival data) from 1975 to 2015 is shown in Figure 2. The R values in Table 1 were compared to each other, with the resultant z-statistics and *p* values shown in Table 2. Figure 3 illustrates the increases in R over time, along with some data from the literature reflecting the increased use of FDG-PET for cancer staging over time [9].

As noted, the data for larynx, lung and bronchus, and prostate cancers are incomplete across the time intervals considered. Therefore, those cancer sites were omitted and the above analysis was repeated for the remaining 15 cancer sites. The results are similar and are demonstrated in Supplementary Table S2.

Using the cancer-specific death rates at 5-, 10-, and 15-years post-diagnosis is an imperfect means to estimate the rate of metachronous metastases, particularly for tumors where patients often succumb due to uncontrolled localized/regional disease or cancer-specific death rates may be relatively low. The above analysis was thus repeated with cancers of the head and neck (oral cavity and pharynx, and larynx) and cervix omitted (i.e., tumor sites where uncontrolled localized/regional disease might more often cause mortality), and the results are similar; i.e., increases in the R values over time. More details are provided in Supplementary Table S3.

Broad changes in clinical care over time limits the ability to ascribe changes seen over time *solely* to improvements in imaging. For example, increases in cancer screening will identify cancers earlier in their natural history, and thus tend to reduce the overall rates of metastases (synchronous plus metachronous). Similarly, increasingly effective adjuvant and therapeutic systemic therapy will tend to decrease the apparent rate of metachronous metastases. Changes in tumor biology/aggressiveness and patient-based factors over time may also impact these observations. See Supplementary Figure S1 for illustrations of some of these issues. Therefore, the above analyses were repeated with several cancer types removed (e.g., breast, melanoma, and colon; where there have been marked changes in screening, and/or effective systemic therapies, over time), and the overall results were again similar.

## 4. Discussion

The data extracted from SEER suggest that there are meaningful associations between the rates of metachronous and synchronous metastases, and that those associations appear to become stronger over time (i.e., apparent increases in the R values). That observation was the same for analyses based on the 5-, 10-, or 15-year survival data.

The reasons for the stronger associations over time are unknown, and might include changes in the biology of tumors (e.g., increasing aggressiveness for some tumors; increasing detection of earlier tumors due to screening, changes in the fraction of virally mediated tumors [e.g., associated with HPV]), shifts in patient demographics (e.g., increasing age), societal changes in behaviors (e.g., tobacco use, and other life-style choices) over time.

As shown in Figure 3, the timing of the increasing association between the rates of metachronous and synchronous metastases is contemporaneous with the increasing use of FDG-PET imaging for the staging of cancer. The Centers for Medicare and Medicaid Services started to formally cover FDG-PET for the initial staging of non-small cell lung cancer in 1998, and its coverage was expanded for initial staging of other cancers in subsequent years. FDG-PET is generally considered to be more accurate and/or sensitive than other imaging modalities for metabolically active cancers utilizing glycolytic pathways [10,11]. That, therefore, suggests that the increasing use of more accurate staging studies, such as FDG-PET, may be (at least in part) related to the observed increasing association between the estimated rates of metachronous and synchronous metastases.

The observed apparent increasing association between the rates of metachronous and synchronous metastases over time in the current analysis was not readily apparent in our prior analyses [3], likely since the prior analyses considered data over a fewer number of years, and with shorter follow-up durations (i.e., Anacak [3] considered 5-year follow-up data from 1973 through 1998, compared to the current analysis that considered 5–15 year follow-up data from 1975 through 2015).

It is tempting to consider changes in the *slopes* (with their 95% CIs) of the regression lines over time. However, interpreting these changes in slope are challenging since they represent complex disease-specific changes in the *absolute* rates of synchronous and metachronous metastases over time due to changes in imaging, screening, and systemic therapies. For example, the rates of synchronous metastases might have declined due to increasing rates of screening (e.g., for cancers of the breast, cervix, and prostate). Rates of apparent metachronous metastases might decrease for diseases where systemic therapies have become much more effective over time (e.g., cancers of the breast, colon, and stomach). Given the complex interactions between these various factors, it is challenging to analyze changes in the slopes of the regression lines over time. Nonetheless, the data points are becoming closer to the regression line and the standard errors are becoming smaller over time (see data points and gray areas around regression lines in Figure 2) consistent with the R values increasing.

Thus, the data is consistent with our hypothesis that the association between the rates of synchronous and metachronous metastases, across different types of cancer, has increased over time. Indeed, by 2015, the pooled data across all cancer types considered suggest that the rate of metachronous metastases is roughly 50% of the rate of synchronous metastases (alternatively stated, roughly two-thirds of all metastases present at the time of diagnosis are clinically detectable at the time of diagnosis). This provides an overall *quantitative* estimate of the utility/limitations of current staging methods. This may help both providers and patients during discussions regarding prognosis and treatment recommendations. Furthermore, this information might assess whether some adjuvant therapies are helpful in delaying or preventing metachronous metastases (as described in detail in the Introduction). These potential utilities in an improved understanding of the association between the rates of synchronous and metachronous metastases exist irrespective of the precise cause of these strengthening associations over time.

R values are increasing over time; however, the *p* values from the Fisher’s z-transformation analysis (that formally compare these R values to each other) are largely >0.05. This likely the reality that the Fisher’s z-transformation analysis is affected by sample size, and we have only ≤18 different data points (i.e., cancer sites) to assess. Thus, the non-significant *p* values (i.e., >0.05; also an arbitrary threshold) should not be taken to totally dismiss our observations.

Our study has several limitations. First, there are inherent inaccuracies in population-based data. However, these large datasets are widely used [12,13], as their size helps to overcome those inaccuracies. Second, the methods used to estimate the synchronous and metachronous distant metastases rate are imperfect. As this same imperfect measure was used over time, we believe that the approach taken was reasonable. Furthermore, we recognize that our assumption that all metachronous distant metastases are present at the time of diagnosis is imperfect (e.g., some of these likely occur due to persistent/recurrent localized/regional disease). Nevertheless, analysis of whole-exome sequencing data of primary and metastatic tumor pairs (including breast, lung, and colorectal cancers) implies that systemic spread can be initiated rapidly following malignant transformation, *often happening 2–4 years before* diagnosis of the primary tumor, thus lending support to our underlying assumption [2].

Third, we also recognize that not all deaths are due to distant metastases. Nevertheless, most deaths from cancer are due to metastases [14], and we did re-conduct the analysis after removing head and neck cancer and cervix cancer from the analysis, and the results were largely unchanged, as noted. Fourth, there exist missing data for larynx, lung and bronchus, and prostate cancers. However, the results remained similar when the analysis was repeated with these diseases omitted. Nonetheless, due to the strong relationship between the rates of synchronous and metachronous metastases for 2015 data, the analysis results remain similar regardless of which cancer sites we omit. Fifth, the statistical comparison of R values with the z-test is suboptimal as the number of data points used to calculate the R values are limited.

Sixth, each tumor site was considered as a single data point; e.g., we did not consider subtypes of different cancers. While it might be instructive to consider different cancer subtypes (e.g., by grade, histology, etc.) within a single tumor location independently, such subtype information was not generally reported by SEER during the decades-long time frame we considered (e.g., HPV status in head and neck cancer and HER-2 status for breast cancer have been reported only since 2010). Nevertheless, some subtype information for a few cancer sites has been reported (e.g., ER/PR status, and patient age [a surrogate for menopausal status within] in breast cancer cases; and tumor histology for lung cancer). The timing of the changes in the estimated distribution of metastases (i.e., synchronous vs. metachronous) is similar amongst the subgroups and the overall disease-site data (see Supplementary File S1).

Seventh, we are unable to conclusively link the observed changes in the degree of association between the estimated rates of synchronous and metachronous distant metastases to changes in imaging. There are no good data on the usage rates of different imaging modalities for most tumor sites, thus limiting site-specific analysis. Nevertheless, there are good data for lung cancer, and our prior analysis suggests that an increase in the fraction of synchronous metastases happened concurrently with the increased use of FDG-PET in these patients [15]. Furthermore, we believe that there is utility in recognizing the increasing association between the estimated rates of synchronous and metachronous metastases, irrespective of the cause of the increasing association. We recognize that the overall quantitative approaches that we have taken are imprecise (based on the nature of the available data), and therefore that the results and statistical analysis might be best considered as largely descriptive.

Some of the above-noted limitations may be minimized in coming years as the SEER data begin to mature following the advent of targeted radiotracers that have revolutionized the staging of certain types of cancer [16]. For example, in prostate cancer, prostate-specific membrane antigen (PSMA)-targeted PET radiotracers demonstrate profoundly improved detection efficiency for sites of metastatic disease relative to prior conventional imaging methods [17]. As that type of success may be recapitulated in other cancer types using other targets amenable to PET radiotracer development, we can prospectively hypothesize that the associations between the rates of synchronous and metachronous metastases may continue to strengthen over time (to the degree that the observations herein described are related to improvements in imaging).

## 5. Conclusions

This analysis suggests that there is an increasing association between the rates of synchronous and metachronous distant metastases over time. The pooled data across all cancer types considered suggest that the rate of metachronous metastases is ≈50% of the rate of synchronous metastases (i.e., ≈two-thirds of all metastases *present* at the time of diagnosis are *clinically detectable* at diagnosis). This overall *quantitative* estimate of the utility/limitations of current staging methods may help both providers and patients during discussions regarding prognosis and treatment recommendations. Furthermore, this information might assess whether some adjuvant therapies are helpful in delaying or preventing metachronous metastases. These potential utilities in an improved understanding of the association between the rates of synchronous and metachronous metastases exist irrespective of the precise cause of these strengthening associations over time.

The exact causes for this increasing association between the rates of synchronous and metachronous distant metastases over time are unknown. However, the timing of the increasing association is somewhat contemporaneous with the broader adoption of more accurate cancer imaging studies (e.g., FDG-PET), suggesting that improved imaging might have played (at least a partial) role in this observed change.

## Figures and Tables

**Table 1:** Regression analysis between the rates of synchronous metastases and each of 5-year, 10-year, and 15-year metachronous metastases across all cancer sites considered.

Year	5-Year Data			10-Year Data			15-Year Data		
	R	Slope (95% CI)	*p*	R	Slope (95% CI)	*p*	R	Slope (95% CI)	*p*
1975	0.59	0.46 (0.10, 0.83)	0.02	0.47	0.37 (−0.03, 0.77)	0.06	0.42	0.34 (−0.08, 0.76)	0.11
1985	0.60	0.56 (0.13, 0.98)	0.01	0.49	0.48 (−0.01, 0.96)	0.05	0.43	0.43 (−0.08, 0.95)	0.09
1995	0.66	0.59 (0.23, 0.94)	0.003	0.58	0.54 (0.14, 0.94)	0.01	0.52	0.50 (0.06, 0.94)	0.03
2005	0.80	0.57 (0.33, 0.80)	<0.001	0.76	0.58 (0.31, 0.85)	<0.001	0.75	0.57 (0.29, 0.85)	<0.001
2015	0.87	0.57 (0.39, 0.74)	<0.001						

**Table 2:** Fisher’s z transformation to assess the difference between pairs of R values derived from different years (*p* values are one-tailed).

Years Being Compared	5-Year Data		10-Year Data		15-Year Data	
	z	*p*	z	*p*	z	*p*
1985 vs. 1975	0.04	0.48	0.07	0.47	0.03	0.49
1995 vs. −1975	0.30	0.38	0.40	0.34	0.34	0.37
2005 vs. −1975	1.09	0.14	1.26	0.10	1.36	0.09
2015 vs. −1975	1.70	0.04				

## Data Availability

The original contributions presented in this study are included in the article/Supplementary Materials. Further inquiries can be directed to the corresponding author.

## Supplementary Materials

The following supporting information can be downloaded at: https://www.mdpi.com/article/10.3390/jcm14082762/s1, Supplementary File S1: Supplement X; Figure S1: Idealized diagram (not to scale) illustrating how the relationship between pathologic stage (pM0 vs. pM1) and clinical stage (cM0 vs. cM1) might be expected to change over time as one moves from older staging tools/imaging (A) to different scenarios including: (B) better imaging tools/imaging; (C) better adjuvant/therapeutic systemic therapy; (D) more screening (earlier detetion), and (E) both better imaging and better adjuvant/therapeutic systemic therapy. The projections of borders between pM0 and pM1, and between cM0 and cM1 in older staging tools/imaging in the panel (A) are represented by dashed lines in panels (B–E). These changes would alter the apparent rates of synchronous and metachronous metastases as shown; Table S1: List of tumor sites along with corresponding rates of synchronous and metachronous metastases; Table S2: Regression analysis between the rates of synchronous metastases and each of 5-year, 10-year and 15-year metachronous metastases (larynx, lung and bronchus and prostate cancers were excluded from the analyses); Table S3: Regression analysis between the rates of synchronous metastases and each of 5-year, 10-year and 15-year metachronous metastases (oral cavity and pharynx, larynx and cervix cancers were excluded from the analyses).
