# Supplementary material for: Increases in the Association Between the Rates of Synchronous and Metachronous Metastases over Time
Source: J Clin Med. 2025 Apr 17;14(8):2762. doi: 10.3390/jcm14082762 (PMC12027837; doi:10.3390/jcm14082762)
Supplement: Supplementary file 1 [file jcm-14-02762-s001.zip › Supplemental figure and tables.pdf]

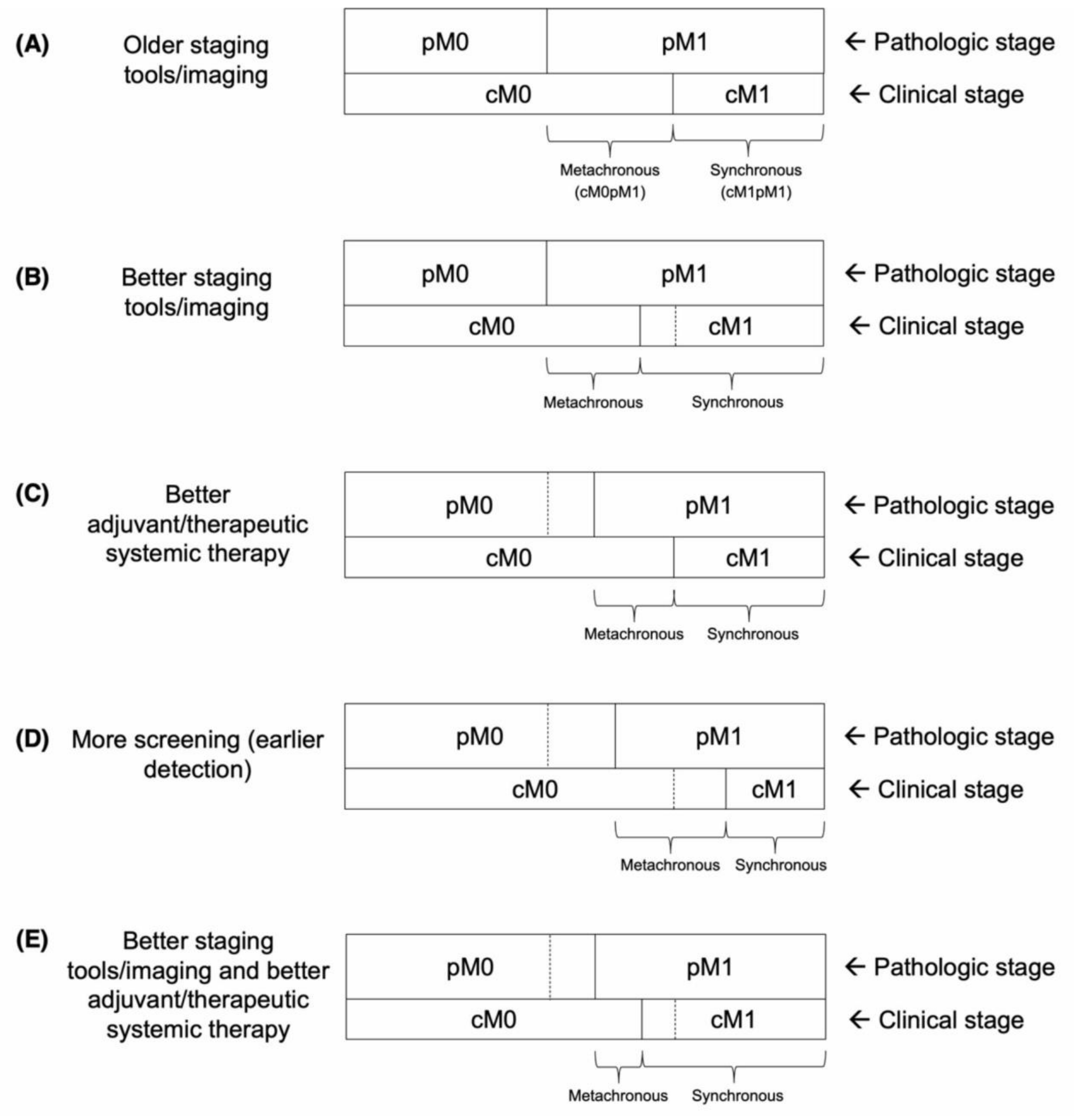

Supplemental Figure S1. Idealized diagram (not to scale) illustrating how the relationship between pathologic stage (pM0 vs. pM1) and clinical stage (cM0 vs. cM1) might be expected to change over time as one moves from older staging tools/imaging (A) to different scenarios including: (B) better imaging tools/imaging; (C) better adjuvant/therapeutic systemic therapy; (D) more screening (earlier detaction), and (E) both better imaging and better adjuvant/therapeutic systemic therapy. The projections of borders between pM0 and pM1, and between cM0 and cM1 in older staging tools/imaging in the panel (A) are represented by dashed lines in panels (B–E). These changes would alter the apparent rates of synchronous and metachronous metastases as shown.

Supplemental Table S1. List of tumor sites along with corresponding rates of synchronous and metachronous metastases.

Tumor site	1975				1985				1995			
	Synch. (%)	5-Year Met. (%)	10-Year Met. (%)	15-Year Met. (%)	Synch. (%)	5-Year Met. (%)	10-Year Met. (%)	15-Year Met. (%)	Synch. (%)	5-Year Met. (%)	10-Year Met. (%)	15-Year Met. (%)
Colon	26	22	25	26	19	19	22	22	21	17	21	22
Rectum and rs. junc.	21	26	32	33	16	24	31	33	16	20	25	27
Esophagus	37	58	58	59	34	56	58	58	31	56	58	60
Oral cav. and phar.	14	31	36	41	10	34	41	46	8	32	40	46
Corpus and uterus	5	6	8	8	10	7	10	10	8	8	10	11
Breast	8	16	24	29	7	14	21	24	6	7	12	15
Cervix uteri	8	21	25	27	10	23	28	31	6	18	22	25
Pancreas	67	31	31	31	61	37	37	37	60	36	37	37
Kidney and renal p.	25	19	24	29	29	14	21	25	23	13	18	23
Stomach	42	40	42	44	39	40	42	42	35	41	43	44
Melanoma of the s.	5	12	17	20	4	9	11	11	3	7	7	8
Urinary bladder	3	23	28	31	3	19	23	27	3	14	19	23
Testis	15	9	12	15	14	2	2	3	11	3	3	3

Thyroid	7	4	5	5	7	3	3	3	6	2	2	2
Soft tissue inc. heart	16	27	33	34	14	21	24	26	17	18	23	25
Larynx	7	30	41	45	5	26	38	43	7	26	37	44
Lung and bronchus	-	-	-	-	-	-	-	-	51	33	36	38
Prostate	-	-	-	-	-	-	-	-	7	5	9	13

**Tumor site**	**2005**				**2015**	
	**Synch. (%)**	**5-Year Met. (%)**	**10-Year Met. (%)**	**15-Year Met. (%)**	**Synch. (%)**	**5-Year Met. (%)**
Colon	21	12	15	17	24	10
Rectum and rs. junc.	16	14	19	21	20	13
Esophagus	39	41	45	46	45	32
Oral cav. and phar.	14	24	31	34	18	15
Corpus and uterus	9	8	10	11	11	6
Breast	6	4	7	9	7	3
Cervix uteri	11	22	25	27	14	16
Pancreas	58	36	37	37	53	34

Kidney and renal p.	16	8	12	15	14	7
Stomach	39	34	37	38	42	25
Melanoma of the s.	4	4	4	4	4	1
Urinary bladder	4	15	19	22	5	12
Testis	9	1	1	2	12	0
Thyroid	5	1	1	1	4	0
Soft tissue inc. heart	18	17	22	24	14	19
Larynx	-	-	-	-	-	-
Lung and bronchus	57	25	29	31	51	23
Prostate	5	3	5	8	9	2

Abbreviations: Synch., Synchronous; Met., Metachronous; Rectum and rs. junc., Rectum and rectosigmoid junction; Oral cav. and phar., Oral cavity and pharynx; Kidney and renal p., Kidney and renal pelvis; Melanoma of the s., Melanoma of the skin; Soft tissue inc. heart, Soft tissue including heart

Supplemental Table S2. Regression analysis between the rates of synchronous metastases and each of 5-year, 10-year and 15-year metachronous metastases (larynx, lung and bronchus and prostate cancers were excluded from the analyses).

**Year**	**5-year data**			**10-year data**			**15-year data**		
	R	Slope (95% CI)	*p*	R	Slope (95% CI)	*p*	R	Slope (95% CI)	*p*
1975	0.63	0.50 (0.13, 0.87)	0.01	0.55	0.42 (0.03, 0.82)	0.04	0.50	0.39 (-0.01, 0.80)	0.06
1985	0.64	0.60 (0.17, 1.04)	0.01	0.57	0.55 (0.07, 1.03)	0.03	0.52	0.51 (0.01, 1.02)	0.05
1995	0.67	0.66 (0.23, 1.10)	0.006	0.61	0.62 (0.14, 1.10)	0.01	0.57	0.60 (0.08, 1.12)	0.03
2005	0.83	0.68 (0.41, 0.95)	< 0.001	0.78	0.68 (0.36, 1.01)	< 0.001	0.76	0.67 (0.33, 1.01)	< 0.001
2015	0.89	0.62 (0.43, 0.82)	< 0.001						

Supplemental Table S3. Regression analysis between the rates of synchronous metastases and each of 5-year, 10-year and 15-year metachronous metastases (oral cavity and pharynx, larynx and cervix cancers were excluded from the analyses).

**Year**	**5-year data**			**10-year data**			**15-year data**		
	R	Slope (95% CI)	*p*	R	Slope (95% CI)	*p*	R	Slope (95% CI)	*p*
1975	0.66	0.53 (0.13, 0.93)	0.01	0.58	0.45 (0.03, 0.88)	0.04	0.55	0.43 (0.00, 0.86)	0.05
1985	0.71	0.67 (0.23, 1.11)	0.006	0.67	0.63 (0.16, 1.10)	0.01	0.64	0.60 (0.12, 1.09)	0.02
1995	0.76	0.69 (0.34, 1.04)	< 0.001	0.74	0.67 (0.31, 1.04)	0.002	0.72	0.66 (0.28, 1.04)	0.002
2005	0.86	0.60 (0.39, 0.82)	< 0.001	0.84	0.62 (0.38, 0.86)	< 0.001	0.83	0.61 (0.36, 0.86)	< 0.001
2015	0.90	0.58 (0.41, 0.75)	< 0.001						
