# Supplementary material for: Increases in the Association Between the Rates of Synchronous and Metachronous Metastases over Time
Source: J Clin Med. 2025 Apr 17;14(8):2762. doi: 10.3390/jcm14082762 (PMC12027837; doi:10.3390/jcm14082762)
Supplement: Supplementary file 1 [file jcm-14-02762-s001.zip › Supplementary File S1.pdf]

Yilmaz, Rowe and Marks

Supplementary File S1: Supplement X

In our primary analysis, each tumor site was considered as a single data point; e.g. subtypes of different cancers were not considered. We herein provide some data from SEER regarding changes over time in the estimated fraction of metastases that are synchronous (i.e. (synchronous)/(synchronous + metachronous) for some subtypes of cancer. As shown in the figures, for both breast cancer and lung cancer, the timing of the changes in the estimated fraction of metastases that are synchronous over time, are subjectively largely similar across tumor subtypes and to the pooled data for that anatomic site. In the graphs shown, the 5- 10- and 15-year data refer to using the 5- 10- and 15-year survival data to estimate the metachronous metastases rates. There is sparse subtype information in SEER over the decades-long time frame we considered in our primary analysis, and thus we cannot do this for all tumor sites considered. Further, the limited subtype data available limit formal analyses.

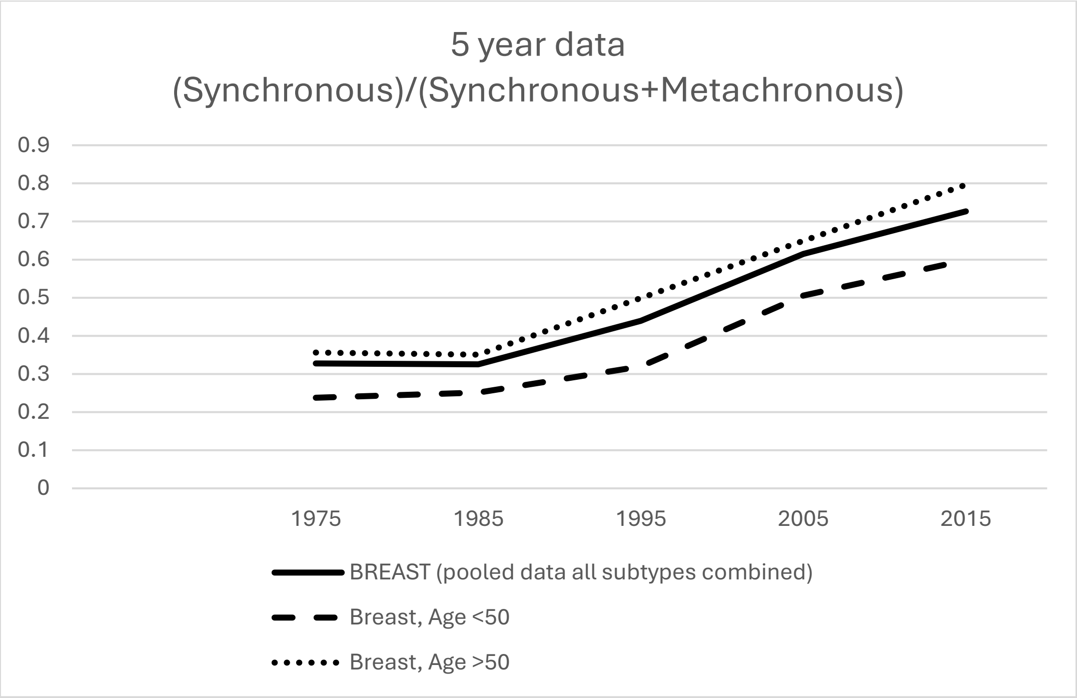

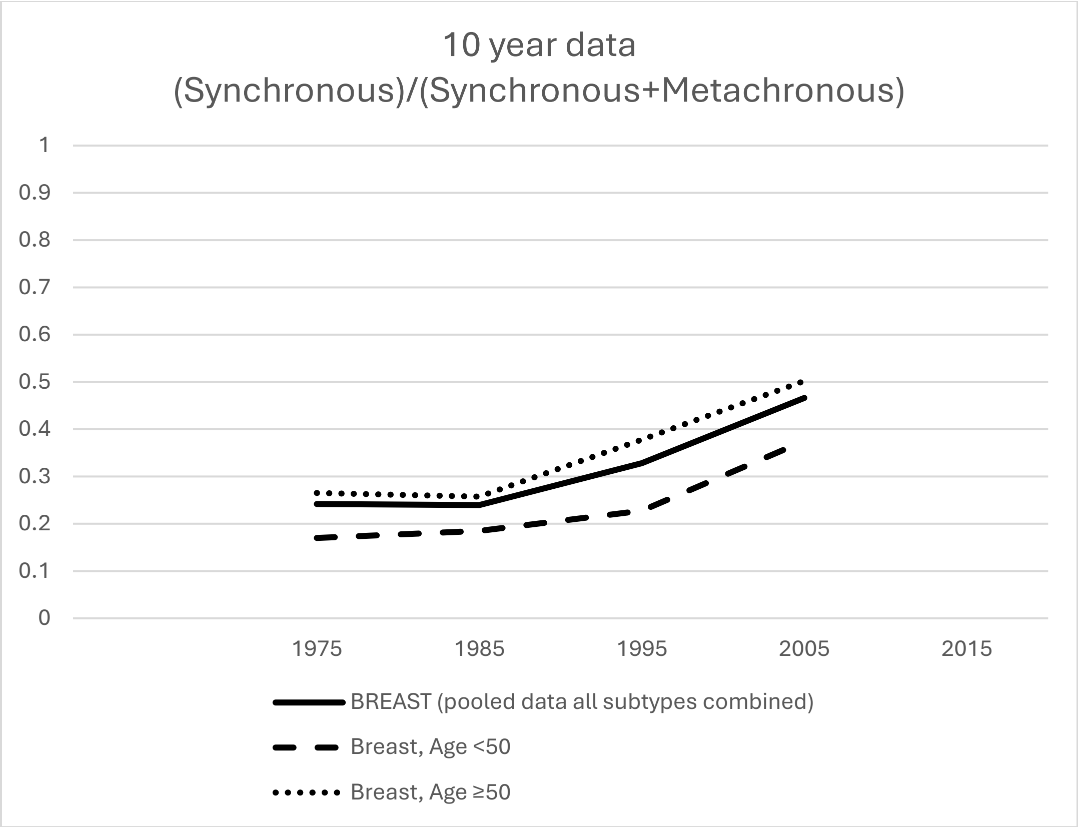

10 year data
(Synchronous)/(Synchronous+Metachronous)
1
0.9
0.8
0.7
0.6
0.5
0.4
0.3
0.2
0.1
0
1975
1985
1995
2005
2015
BREAST (pooled data all subtypes combined)
Breast, Age <50
Breast, Age ≥50

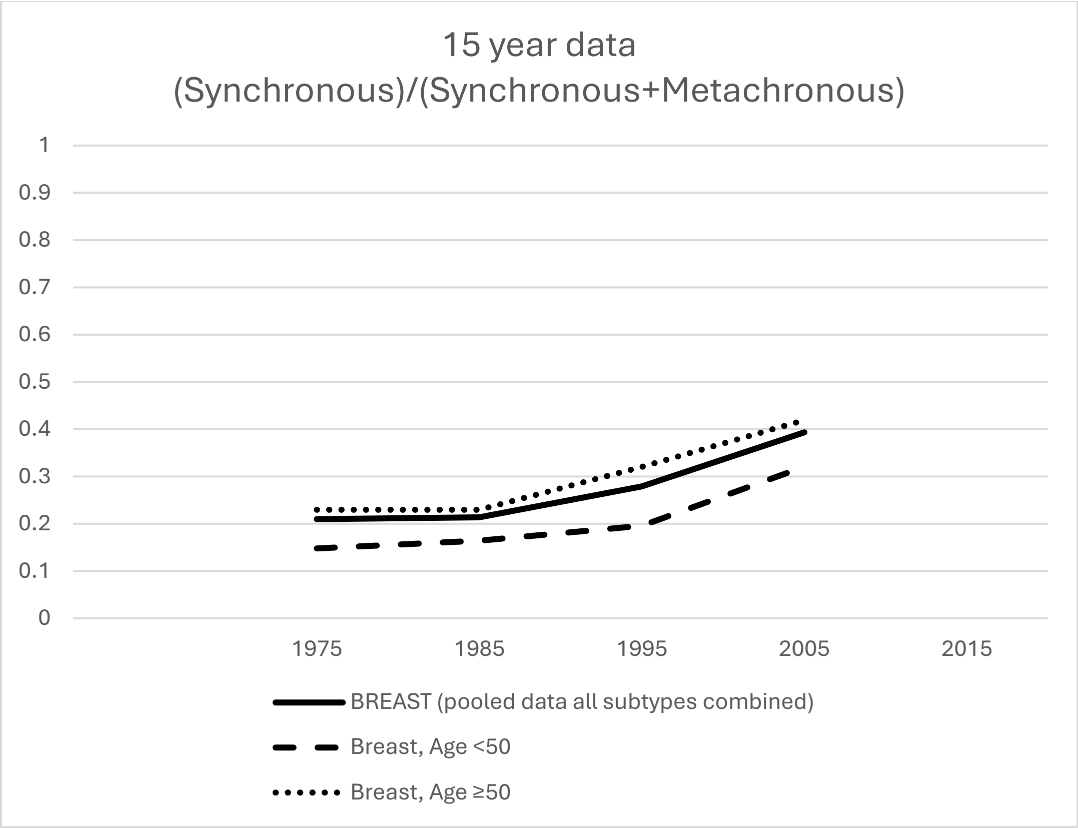
15 year data
(Synchronous)/(Synchronous+Metachronous)
1
0.9
0.8
0.7
0.6
0.5
0.4
0.3
0.2
0.1
0
1975
1985
1995
2005
2015
BREAST (pooled data all subtypes combined)
Breast, Age <50
Breast, Age ≥50

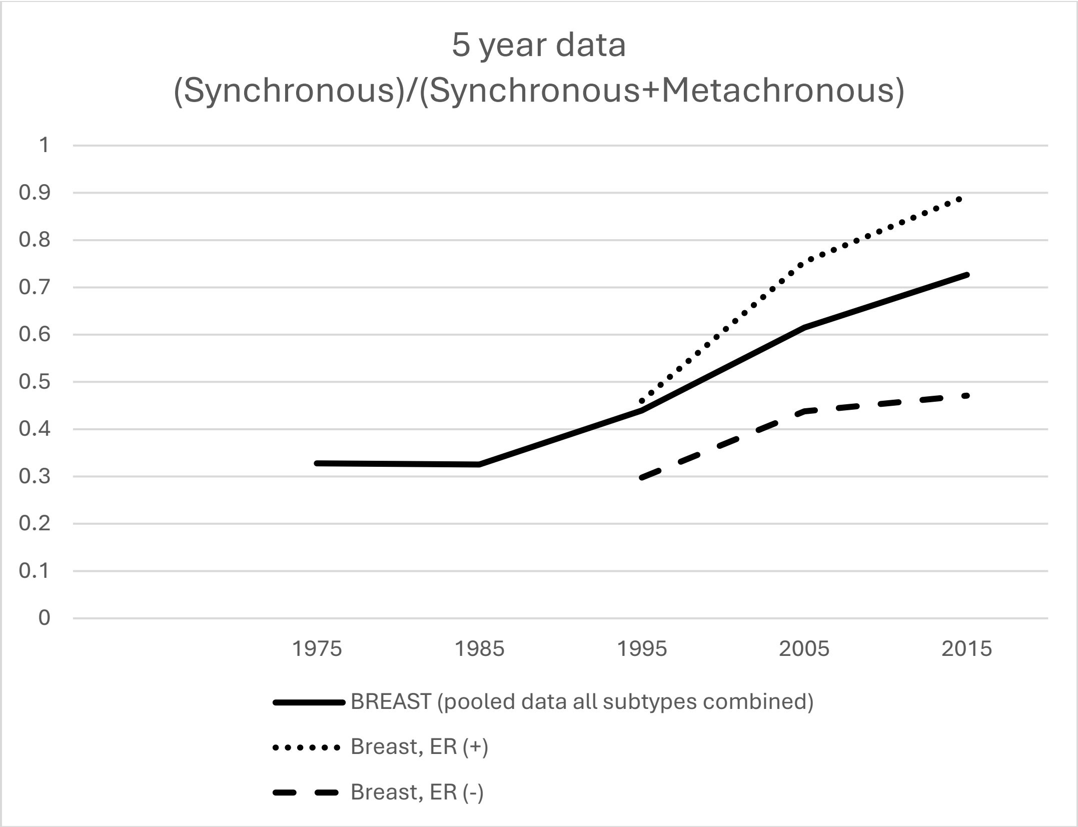
5 year data
(Synchronous)/(Synchronous+Metachronous)
1
0.9
0.8
0.7
0.6
0.5
0.4
0.3
0.2
0.1
0
1975
1985
1995
2005
2015
BREAST (pooled data all subtypes combined)
Breast, ER (+)
Breast, ER (-)

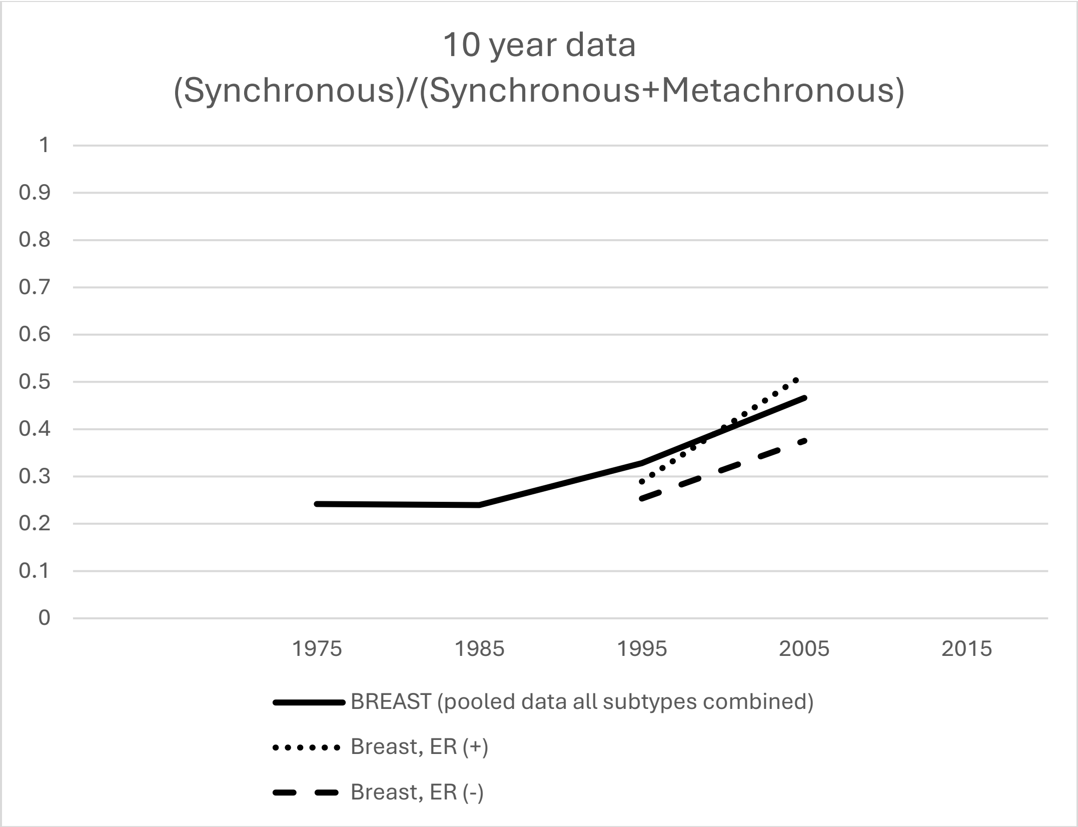
10 year data
(Synchronous)/(Synchronous+Metachronous)
1
0.9
0.8
0.7
0.6
0.5
0.4
0.3
0.2
0.1
0
1975
1985
1995
2005
2015
BREAST (pooled data all subtypes combined)
Breast, ER (+)
Breast, ER (-)

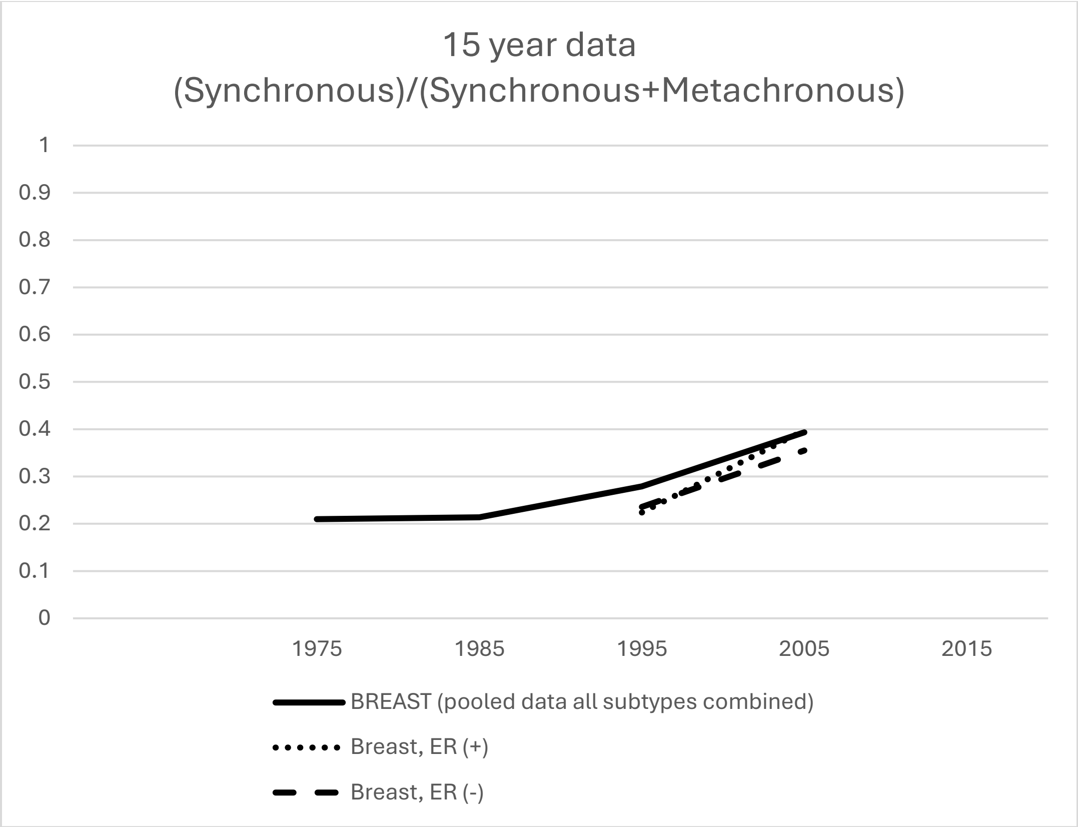
15 year data
(Synchronous)/(Synchronous+Metachronous)
1
0.9
0.8
0.7
0.6
0.5
0.4
0.3
0.2
0.1
0
1975
1985
1995
2005
2015
BREAST (pooled data all subtypes combined)
Breast, ER (+)
Breast, ER (-)

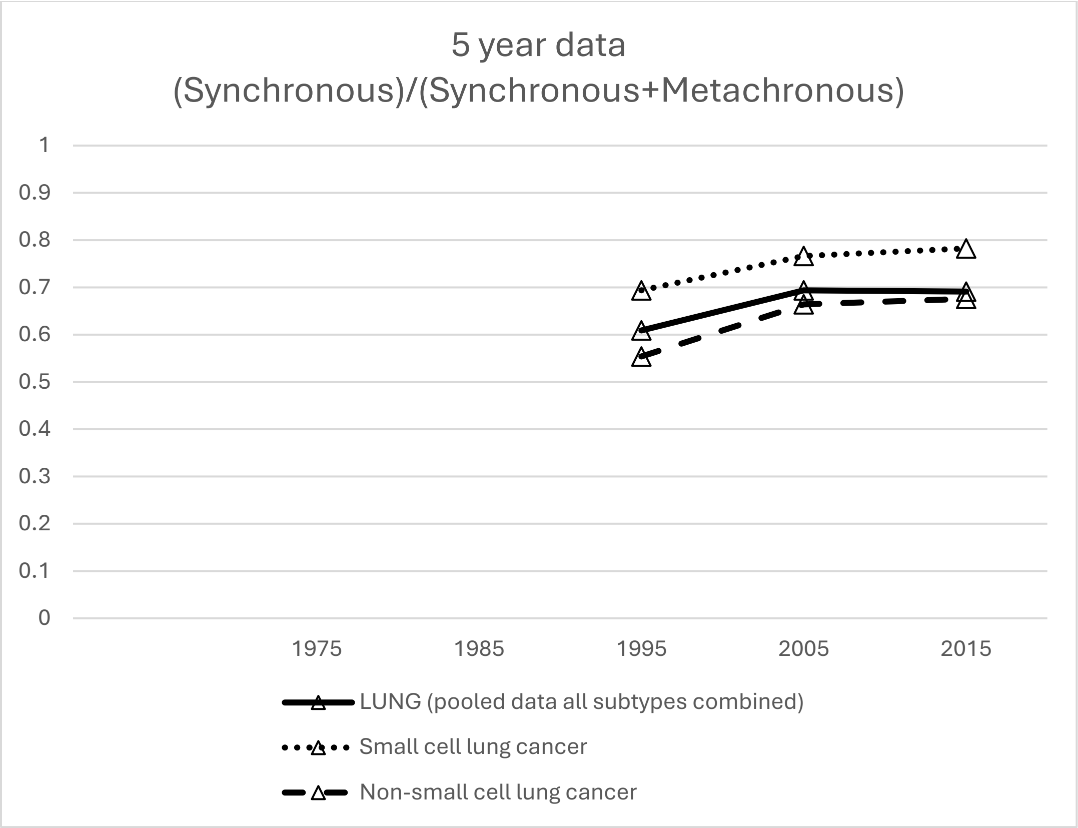
5 year data
(Synchronous)/(Synchronous+Metachronous)
1
0.9
0.8
0.7
0.6
0.5
0.4
0.3
0.2
0.1
0
1975
1985
1995
2005
2015
LUNG (pooled data all subtypes combined)
Small cell lung cancer
Non-small cell lung cancer

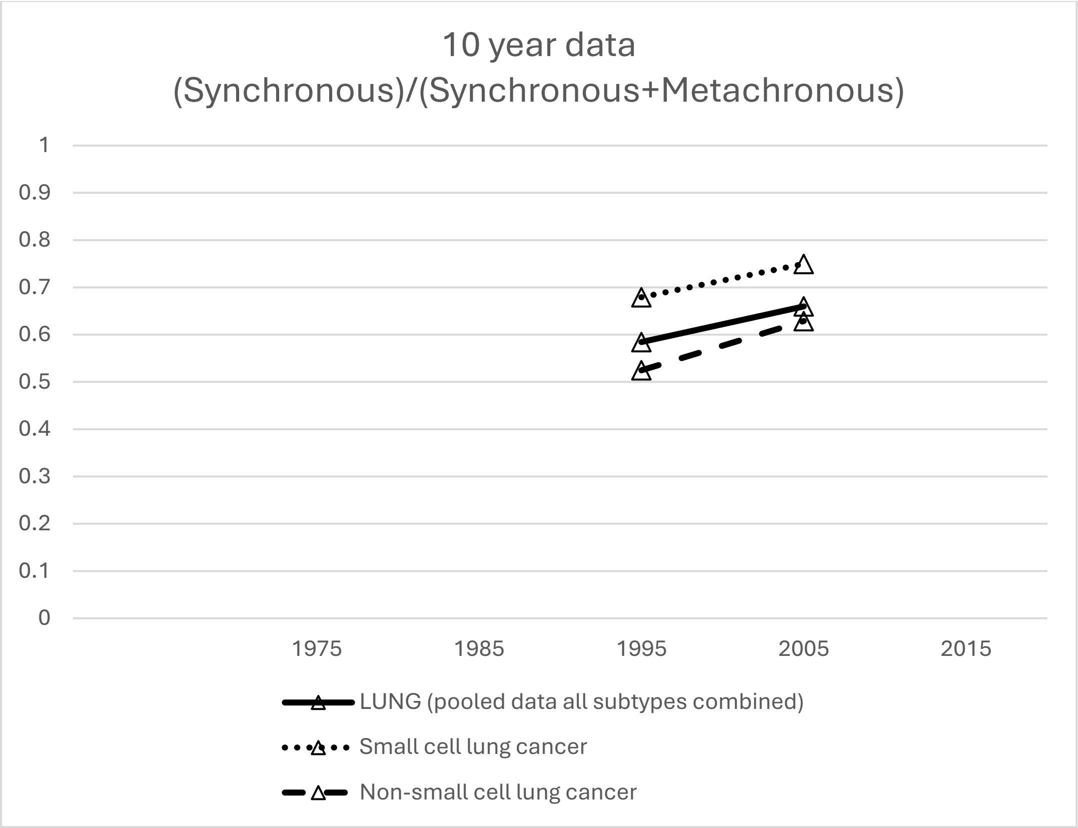
10 year data
(Synchronous)/(Synchronous+Metachronous)
1
0.9
0.8
0.7
0.6
0.5
0.4
0.3
0.2
0.1
0
1975
1985
1995
2005
2015
LUNG (pooled data all subtypes combined)
Small cell lung cancer
Non-small cell lung cancer

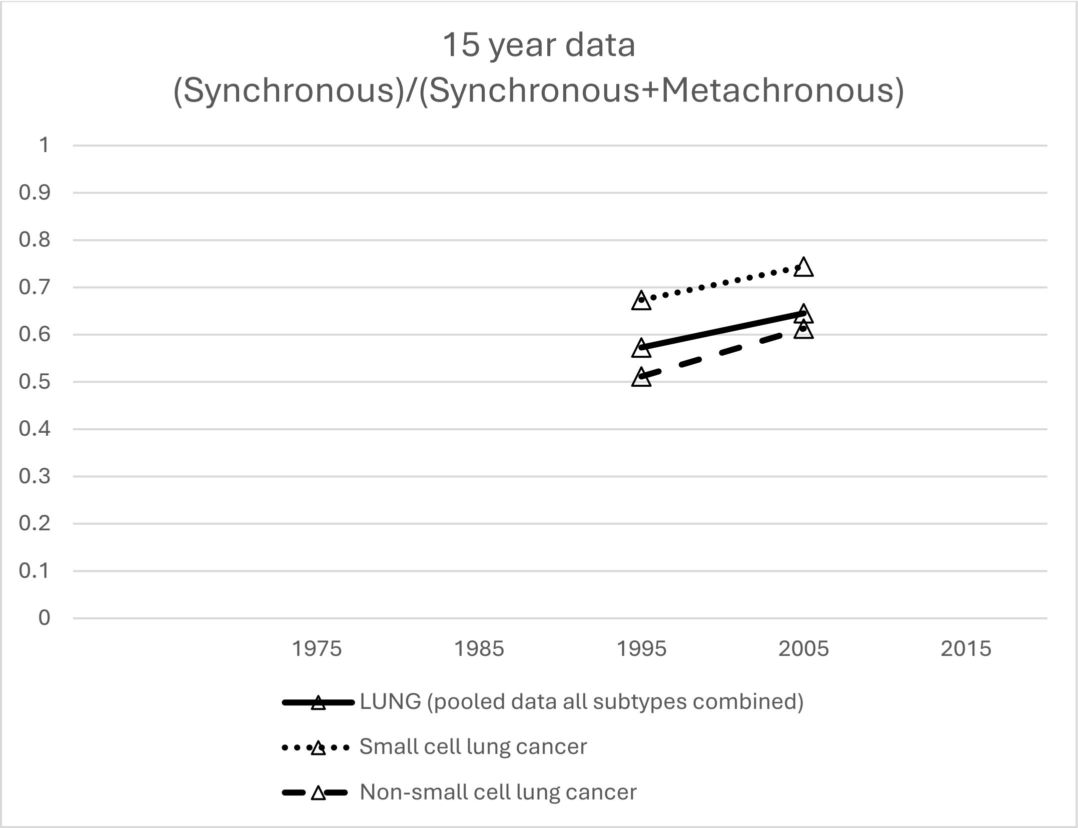
15 year data
(Synchronous)/(Synchronous+Metachronous)
1
0.9
0.8
0.7
0.6
0.5
0.4
0.3
0.2
0.1
0
1975
1985
1995
2005
2015
LUNG (pooled data all subtypes combined)
Small cell lung cancer
Non-small cell lung cancer
